# Phantom Quantification of Magnetoencephalography Source Imaging Distortion Caused by Deep Brain Stimulation

**DOI:** 10.3390/brainsci16060554

**Published:** 2026-05-22

**Authors:** Saar Kariv, Jeong Woo Choi, Amy L. Proskovec, Mahak Virlley, Tyrell Pruitt, Nader Pouratian, Elizabeth M. Davenport

**Affiliations:** 1 Department of Neurological Surgery, The University of Texas Southwestern Medical Center, Dallas, TX 75390, USA; saar.kariv@utsouthwestern.edu (S.K.); jeongwoo.choi@utsouthwestern.edu (J.W.C.); nader.pouratian@utsouthwestern.edu (N.P.); 2 Department of Radiology, The University of Texas Southwestern Medical Center, Dallas, TX 75390, USA; amy.proskovec@utsouthwestern.edu (A.L.P.); mahak.virlley@utsouthwestern.edu (M.V.); tyrell.pruitt@utsouthwestern.edu (T.P.); 3 Magnetoencephalography Center of Excellence, University of Texas Southwestern Medical Center, Dallas, TX 75390, USA; 4 Department of Neurology, The University of Texas Southwestern Medical Center, Dallas, TX 75390, USA

**Keywords:** magnetoencephalography, deep brain stimulation, DBS, DBS noise, DBS artifacts, MEG source imaging, DBS-related artifacts

## Abstract

**Highlights:**

**What are the main findings?**

- Bipolar DBS does not significantly impair MEG dipole estimation in a phantom model.
- cHPI signal quality remains stable and reliable during bipolar DBS in a phantom model.

**What are the implications of the main findings?**

- MEG source-level reconstruction is feasible and may remain reliable during concurrent bipolar DBS.
- This study provides a framework for quantitatively assessing MEG source-level distortions caused by DBS or other implanted or external devices.

**Abstract:**

**Objective:** Previous studies of deep brain stimulation-related (DBS) artifacts in magnetoencephalography (MEG) have largely focused on the sensor level. In contrast, far less is known about their effects at the source level, where neuroscientific interpretations are typically derived. This study aims to quantify how DBS artifacts distort source-level MEG imaging. **Methods:** The study used a phantom-based experimental setup to assess dipole-fitting accuracy while systematically varying the stimulation amplitude, DBS electrode configuration, and the distance between the dipole and the DBS electrode. **Results:** Dipole location, angle, and amplitude errors remained within modest ranges, with the largest location and angle errors occurring at 5 mA ring-electrode stimulation (6.19 mm and 8.31 deg, respectively) and the largest amplitude errors at 15 mA ring electrodes (13.05 nAm). Location and angle errors increased significantly as the dipole moved closer to the DBS electrode, while amplitude error showed no such relationship. Continuous head position indicator coil signal quality remained stable and reliable at DBS on condition, compared to DBS off. **Conclusions:** The stimulation itself does not significantly impair MEG dipole estimation, as fitting errors are similar with DBS on and off. The study introduces a quantitative framework to systematically assess DBS-related distortion via dipole-fitting error, which can also be extended to evaluate noise from other implanted or external devices.

## 1. Introduction

Deep brain stimulation (DBS) is an established neuromodulation technique used in the treatment of an increasing number of neurological and psychiatric disorders [1]. Despite its growing clinical application, the mechanisms underlying its therapeutic effects remain only partially understood. Functional brain imaging provides a powerful approach to studying how the brain operates and how its function is altered by different treatment modalities, which can guide therapeutic optimization and innovation. Magnetoencephalography (MEG) is a noninvasive functional brain imaging technique that records the magnetic fields generated by neuronal activity [2,3,4,5,6,7,8]. It offers sub-millisecond temporal resolution and full cortical coverage using an array of usually more than 250 magnetic sensors. Sophisticated source reconstruction algorithms allow estimation of cortical activity at thousands of points across the cortex, making MEG an increasingly valuable tool for neuroscientists [9,10].

MEG relies on detecting brain signals that are on the order of femtoteslas (10^−15^ T)—10 to 100 million times weaker than Earth’s static magnetic field [2]. This is achieved using finely tuned magnetic sensors placed inside a magnetically shielded room (MSR), which attenuates external magnetic fields. However, the same sensitivity makes MEG extremely vulnerable to electromagnetic interference [11,12].

DBS stimulators contain ferromagnetic components whose motion induces low-frequency artifacts [13,14]. Additionally, turning the stimulator on generates strong electromagnetic fields that can induce severe artifacts in MEG recordings, reducing the signal-to-noise ratio (SNR) and complicating the use of concurrent MEG in DBS research [14,15]. Thus, applying MEG to study DBS poses a significant engineering challenge.

Several research groups have investigated how DBS affects MEG signals and explored methods to mitigate these artifacts [13,14,15,16,17,18,19,20,21,22]. These works have greatly advanced our understanding of DBS-induced artifacts in MEG, allowing scientists to uncover some of the mechanisms by which DBS works. Importantly, these studies have consistently shown that DBS artifacts consist of two primary components: (1) high-amplitude artifacts centered around the stimulation frequency, arising directly from the stimulation current; and (2) low-frequency artifacts generated by movement of the ferromagnetic materials composing the DBS system [14,15,16]. Nonetheless, important gaps remain.

MEG sensors are positioned outside the head, approximately 3–4 cm from the brain, and therefore, provide only coarse information about the origin of neural signals. Source reconstruction employs advanced algorithms to estimate the specific locations of these signals within the brain, achieving spatial resolutions on the order of a few millimeters in the absence of DBS [2].

Most prior studies have focused on the sensor level, characterizing the impact of DBS artifacts on the sensor data [14,15,16,17,20]. Much less is known about their influence at the source level, after source reconstruction within the brain, where neuroscientific conclusions are typically drawn.

The objectives of this study are therefore to:

1. Quantitatively characterize how DBS artifacts distort source-level MEG imaging.
2. Determine if and how source-level distortion depends on key parameters such as the distance between the source and the active DBS electrode, the stimulation amplitude, and the DBS electrode configuration.
3. Assess how DBS artifacts affect the quality of continuous head position indicator (cHPI) system signals.
4. Suggest a platform for future studies to test DBS artifacts in MEG and evaluate artifact reduction methods.

To achieve these goals, we constructed a phantom-based experimental setup to utilize during concurrent MEG. Using this platform, we evaluated dipole fitting accuracy under varying conditions of source-to-DBS electrode distance, stimulation amplitude, and DBS electrode configuration. Importantly, in this work, we isolate and focus exclusively on the first component described above: the high-amplitude artifacts arising directly from the stimulation current. This controlled approach enables a systematic investigation of this specific component and provides a foundation for future studies that incorporate the second component (see details below).

## 2. Methods

### 2.1. MEG System

All recordings were performed using a MEGIN TRIUX 306-channel MEG system (MEGIN Oy, Espoo, Finland; 204 planar gradiometers and 102 magnetometers), with the gantry angle set to 60 degrees. Data were sampled at 3000 Hz with an acquisition bandpass of 0.1–900 Hz. Head-position indicator (HPI) coil frequencies were adjusted from the manufacturer’s default to 457, 507, 587, and 617 Hz in order to mitigate excessive noise and interference with localization.

### 2.2. Phantom

A dry phantom (MEGIN Oy, Espoo, Finland) was used in the experiment. A phantom is a physical object created to mimic brain tissue and activity and, in this case, create dipolar electromagnetic signals to evaluate MEG systems. Specifically, this phantom, designed for system calibration and performance evaluation, contains 32 dipoles. Only one dipole can be activated at a time. The phantom is housed in a hemispheric plastic shell with a radius of 87.5 mm. HPI coils are embedded in the phantom at 4 equidistant points along the perimeter.

To investigate the effect of DBS artifacts as a function of distance from the stimulation source, we selected 10 dipoles distributed across the phantom: 3 left, 3 right, 2 anterior, and 2 posterior (with respect to the phantom orientation). Each dipole was activated at 20 Hz with a 100 nAm peak-to-peak amplitude, simulating weak brain activity.

### 2.3. Experimental Setup

**Figure 1:**
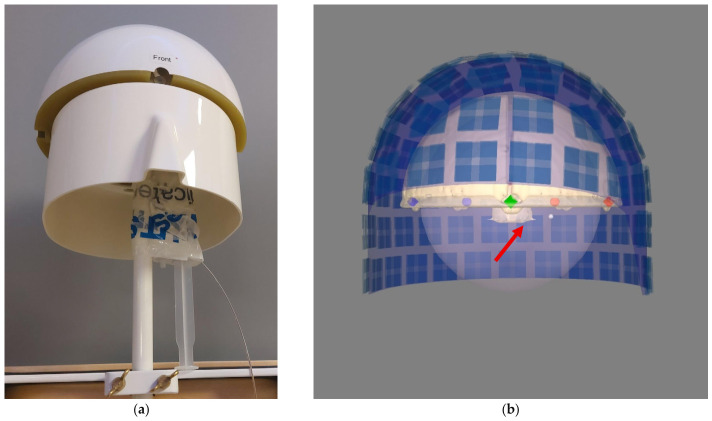
The phantom setup. (**a**) Experimental setup showing the phantom, syringe, front HPI coil, and lead prior to placement inside the MEG system. (**b**) 3D illustration of the phantom and MEG sensor array. The white dot (red arrow) indicates the syringe tip location.

The DBS lead was inserted into a syringe filled with conductive EEG gel, which was fixed to the phantom’s pole with tape (see Figure 1a). The external stimulator was positioned outside the MSR and connected to the system through a conduit in the MSR wall, allowing isolation of the first DBS artifact component described above, which arises directly from the stimulation current.

Stimulation was delivered using an external stimulator (g.ESTIMPRO, g.tec medical engineering GmbH, Schiedlberg, Austria). Stimulation parameters were frequency—130 Hz, pulse width—60 µs, and active recharge waveform (a positive rectangular pulse followed by a negative pulse). The stimulator was connected via a dedicated cable (Threshold NeuroDiagnostics, Minoa, NY, USA, Item Number 810334-0), which linked the device to the Medtronic test cable (Medtronic, Minneapolis, MN, USA). A Medtronic DBS lead, extension cable, and test cable (models B33015-42, B3400040 or B3400060, and B31050, respectively) were used.

### 2.4. Experimental Conditions

Recordings were acquired under seven conditions with varying stimulation amplitude, electrode settings, and cHPI status (see Table 1).

**cHPI Signal Quality with Concurrent Stimulation**

HPI coils are typically activated at the beginning of a scan to determine head position in the scanner. cHPI is the continuous use of the HPI signal, which is used to correct for motion, making it a critical feature for future evaluations of patients with movement disorders. The phantom has four HPI coils embedded on the anterior, posterior, left, and right aspects (Figure 1a shows the front coil).

Therefore, in one experimental condition, the cHPI system was enabled concurrently with stimulation (5 mA, bipolar ring contacts). The goal was twofold: (1) to assess cHPI signal quality during DBS and (2) to evaluate dipole fitting accuracy when both cHPI and DBS were active. cHPI signal quality was quantified using the cHPI SNR computed with the mne.chpi.compute_chpi_snr() function in MNE Python. Since the phantom is stationary, its estimated position (derived from the cHPI signal) should remain constant over time. Thus, instability or excessive variability in the calculated phantom’s coordinates would indicate reduced cHPI signal quality. Therefore, to further assess cHPI quality and complement the SNR measurements, we compared head position parameters—quaternions (q1, q2, q3), translations (x, y, z), and goodness of fit (gof)—between DBS-on and DBS-off conditions.

### 2.5. Data Processing

We used MNE Python [23] to process the data (MNE version 1.11.0). All 306 sensors (gradiometers and magnetometers) were used for this analysis (no sensors were excluded). After loading the data (.fif) file, we used mne.find_events() to detect all events. Each event corresponds to a dipole activation instance and is registered in the stim channel (“SYS201”). Once we had a list of all events per dipole, we band-pass filtered the raw data between 1 and 50 Hz using raw.filter() (stopband attenuation 53 dB and −6 dB cutoff frequency of 56.25 Hz with default function parameters; see documentation for details). We then epoched the data based on the detected events. In some dipoles, the first, second, third, and last events were contaminated by signal “jumps”, likely corresponding to the “switching transient” previously described in [24]. These events were therefore excluded from the analysis for all dipoles. After exclusion, an average of 63.79 ± 6.84 (SD) valid events were included per dipole. The number of valid events per dipole did not differ significantly across conditions (Friedman test, *p* = 0.4).

Next, we created a sphere conductor model by using the function mne.make_sphere_model() centered at the origin of the head coordinate frame with a head radius of 80 mm. The sphere model contained the locations of all included dipoles.

Source estimates were performed by fitting current dipoles using the mne.fit_dipole() function. For covariance estimation, we used a 50 ms window preceding each event onset. All events for a given dipole were then averaged, and a single time point at t = 36 ms—corresponding to the peak of the 20 Hz sinusoidal activity—was selected for dipole fitting. By the end of this process, each of the 10 dipoles had a corresponding estimated dipole. Notice that no noise-cleaning algorithms were applied to the data for this analysis. To compute the power spectral density (PSD) across conditions, we used the raw.compute_psd() function (with default parameters; see documentation for details).

### 2.6. Dipole Fitting Error Measurement

We quantified dipole fitting error in terms of location, orientation, and amplitude of each of the evaluated dipoles, using methods similar to those previously published [25]. The phantom setup enables precise error estimation because the true characteristics of each dipole (i.e., location, orientation, and amplitude) are known and can be directly compared to the fitted solution. To this end, we used the function mne.dipole.get_phantom_dipoles(), which gives the position and orientation of each of the 32 dipoles.

The errors were calculated accordingly:

$${ERROR}_{location}=\sqrt{\left({\bar{loc}}_{actual}-\;{\bar{loc}}_{fitted}\right)\cdot \left({\bar{loc}}_{actual}-\;{\bar{loc}}_{fitted}\right)}$$

$${ERROR}_{orientation}=arccos(abs\left(\left({\bar{ori}}_{actual}\cdot \;{\bar{ori}}_{fitted}\right)\right))$$

$${ERROR}_{amplitude}={Amp}_{actual}-{Amp}_{fitted}$$

where ${\bar{loc}}_{actual}$ and ${\bar{loc}}_{fitted}$ are the dipole’s actual and fitted location vectors, ${\bar{ori}}_{actual}$ and ${\bar{ori}}_{fitted}$ are the dipole’s actual and fitted orientation vectors, and ${Amp}_{actual}$ and ${Amp}_{fitted}$ are the dipole’s actual and fitted amplitude scalars.

### 2.7. Measurement of Distance from the Dipole to the Active DBS Electrode

Prior to data acquisition, the location of the syringe tip was recorded using the Polhemus digitization system relative to the phantom (Figure 1b). During phantom preparation, the distal end of the DBS lead was inserted 4 cm beyond the syringe tip. Using the known syringe tip coordinates, the insertion depth, and the electrode contact geometry, we estimated the DBS electrode position within the phantom’s coordinate system. For simplicity, the active DBS electrode location was represented by a single point, which was defined as the midpoint between the upper and lower ring DBS electrodes for all conditions.

## 3. Results

### 3.1. Sensor-Level PSD

**Figure 2:**
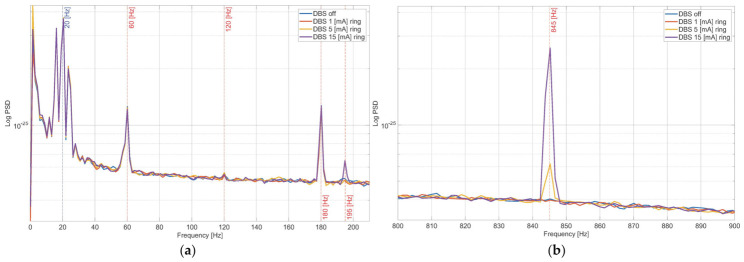
Sensor-level PSD averaged across all sensors, comparing DBS-off and DBS-on (5, 10, and 15 mA, ring contacts). (**a**) 0–200 Hz. (**b**) 800–900 Hz, showing a prominent stimulation artifact peak at 845 Hz.

We first examined the sensor-level PSD to characterize DBS-related noise. Figure 2 shows the sensor-level PSD averaged across all sensors, comparing DBS-off with DBS-on at amplitudes of 1, 5, and 15 mA (ring DBS electrodes with no cHPI). The stimulation induced artifact occurrence at frequencies centered on 130/2 + 130·i Hz (for i = 1, 2, 3…). The artifact magnitude increased with stimulation amplitude (Figure 2b): At 1 mA, no clear artifact spike was observed, whereas distinct peaks appeared at both 5 mA and 15 mA.

The 20 Hz dipolar activity was consistently visible under both DBS-off and DBS-on conditions, with comparable power levels (Figure 2a).

Importantly, the low-frequency range (<50 Hz) remained largely preserved across all DBS-on conditions, showing no added noise relative to DBS-off (Figure 2a). This observation aligns with previous reports suggesting that low-frequency DBS artifacts primarily arise from movement of ferromagnetic extensions (i.e., the second DBS artifact component described above)—factors not present in this setup [13,14,15].

### 3.2. Dipole Fitting Errors

**Figure 3:**
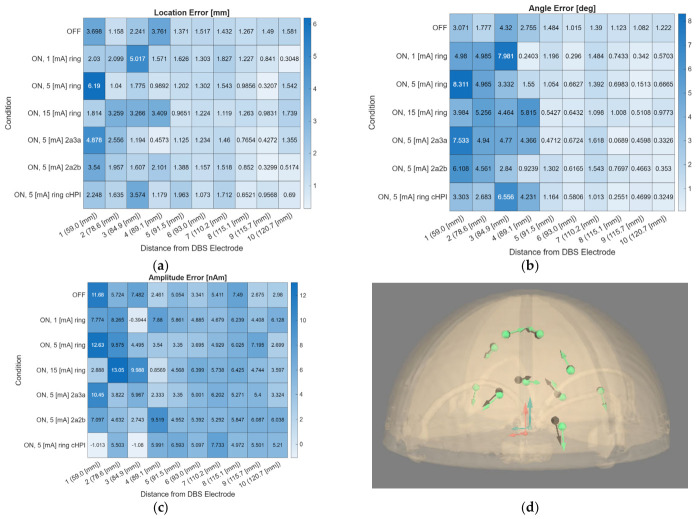
Dipole estimation error. Three tables display the dipole location error (**a**), angle error (**b**), and amplitude error (**c**), with each column representing a dipole with the labeled distance from the DBS electrode and each row representing a different experimental setting. (**d**) Actual (black) vs. fitted (green) dipoles plotted inside 3D visualization of the phantom (seen here for the DBS amplitude, 5 mA ring-electrode condition).

We next examined dipole fitting errors for each assessed dipole. Figure 3 shows the errors in location (Figure 3a), orientation (Figure 3b), and amplitude (Figure 3c), respectively, across experimental conditions (*y*-axis) and ordered by distance from the DBS electrode (*x*-axis).

**Figure 4:**
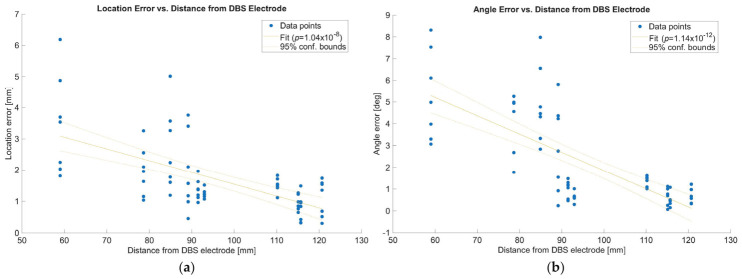
Regression analysis between the dipole’s distance from the DBS electrode and its location (**a**) and angle (**b**) errors, showing a significant error increase as the distance from the DBS electrode decreases.

The location error ranged from 0.30 to 6.19 mm, with the highest error occurring with “5 mA ring electrode” DBS settings. The angle error ranged from 0.069 to 8.31 deg, with the highest error also occurring with “5 mA ring electrode” DBS settings. The amplitude error ranged from −1.08 to 13.05 nAm, with the highest error occurring with “15 mA ring electrode” DBS settings. A significant regression was observed between the dipole’s distance from the DBS electrode and its location and angle errors (Figure 4): as the dipole’s distance decreased, the magnitude of both errors increased (adjusted r^2^ = 0.375, β = −0.037, *p* = 1.04 × 10^−8^; and adjusted r^2^ = 0.52, β = −0.083, *p* = 1.14 × 10^−12^, respectively). However, no significant relationship was found with the amplitude error (*p* = 0.11).

Differences in error amplitudes between different DBS amplitudes, electrode configurations, or cHPI status were not statistically distinguishable (Friedman test; see Table 2, Table 3 and Table 4).

### 3.3. cHPI Signal Quality

**Figure 5:**
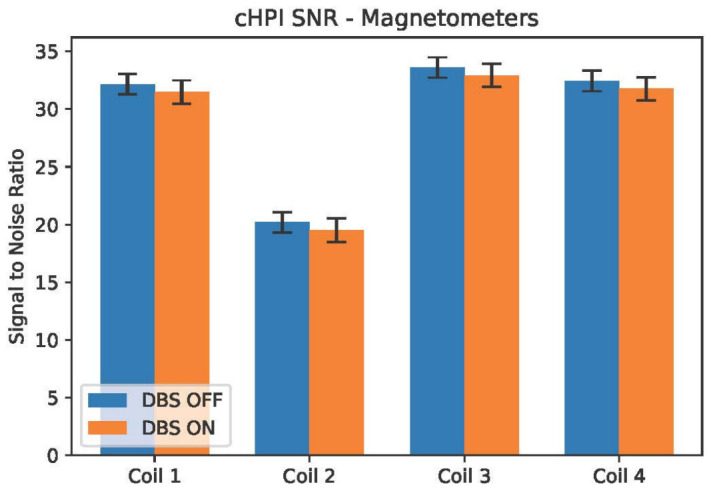
Bar plot of SNR for each cHPI coil under DBS-off and DBS-on conditions.

**Figure 6:**
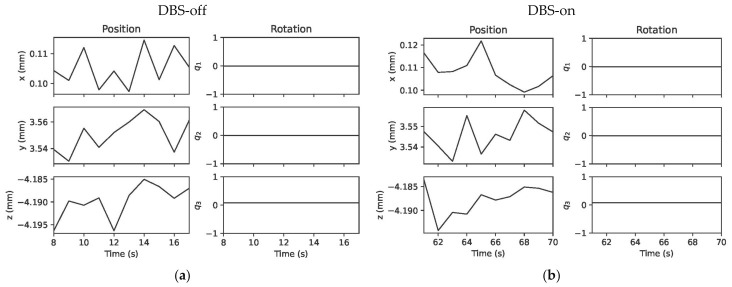
Graphs of phantom positional parameters (linear displacement and rotation) for DBS-off (**a**) and DBS-on (**b**).

Although stimulation leads to a reduction in cHPI SNR, this reduction does not translate into significant measurement inaccuracies. In the DBS-off condition, the cHPI system consistently showed higher magnetometer SNR compared to the DBS-on condition, as demonstrated graphically (Figure 5). The phantom position parameters remained stable across all evaluated measures—linear displacement, rotation, and gof—as confirmed by graphical analysis with both DBS-off and DBS-on (Figure 6) and statistical analyses (Supplementary Material).

## 4. Discussion

Previous studies have employed MEG to investigate the neuromodulatory effects of DBS [26,27,28,29]. For instance, Spooner et al. demonstrated that MEG could aid in optimizing DBS parameters in patients with Parkinson’s disease (PD) by recording sensorimotor evoked responses to single pulses delivered through different DBS electrodes [26]. Wang et al. reported that DBS enhances high-beta/low-gamma corticocortical connectivity, thereby improving network efficiency and modularity [27]. Pauls et al. showed that PD patients have a lower sensorimotor beta burst rate compared to healthy controls, which subthalamic nucleus DBS (STN-DBS) normalizes [28]. Despite the knowledge gained from these studies, DBS still introduces substantial artifacts into MEG recordings, a challenge that warrants further investigation.

Stimulation introduces spectral artifacts at specific frequencies (i.e., the first DBS artifact component) [14,15,19]. Interestingly, unlike previous studies, our analysis revealed that artifacts are centered at subharmonics of the stimulation frequency (130/2 + 130·i for i = 1, 2, 3, …), rather than at the stimulation frequency itself. This shift may reflect differences in experimental setup or signal propagation mechanisms. Importantly, and in line with earlier findings, oscillations below 50 Hz remained unaffected by stimulation, as shown by the consistent detection of 20 Hz dipolar activity across all stimulation amplitudes. Moreover, we found that dipole fitting accuracy is not significantly influenced by DBS condition (off vs. on), stimulation amplitude, electrode configuration, or cHPI status.

A significant increase in location and angle fitting errors was observed with the decrease in the dipole’s distance from the DBS electrode, indicating that source imaging may be less reliable in regions proximal to the electrode. However, the relationship between the degree of source imaging distortion and electrode distance requires further investigation.

Notably, the absolute magnitude of these errors is low across all conditions, even at the highest stimulation amplitude (15 mA)—a somewhat surprising but encouraging result. With respect to cHPI, although a statistically significant reduction in magnetometer SNR is observed in the DBS on condition, this reduction does not compromise phantom position tracking. Finally, both graphical and statistical analyses confirmed stable head position estimates across DBS-off and DBS-on conditions, supporting the feasibility of using cHPI during DBS studies with MEG.

Taken together, these findings demonstrate that the stimulation (i.e., the first DBS artifact component) does not substantially degrade MEG-estimated dipoles, with dipole fitting errors remaining comparable between DBS-off and DBS-on conditions. We introduce a quantitative framework for systematically assessing DBS-induced distortion by measuring dipole fitting error across multiple parameters. This approach may also be applied to evaluate noise effects from other implanted or external devices.

## 5. Limitations

Our phantom setup did not incorporate movement of ferromagnetic materials (i.e., the second DBS artifact component). Prior studies have shown that phantom motion and attached ferromagnetic materials (e.g., extension wires) can introduce low-frequency noise (<50 Hz), a band that is physiologically critical [13,14,15,19]. In this work, introducing this DBS artifact component will likely distort the source reconstruction solution to a degree to be investigated in future studies.

Introducing controlled movement in our setup would likely add this low-frequency noise and could increase the magnitude of measured errors. However, studying MEG artifacts generated by the movement of a ferromagnetic material is a complex engineering challenge that is outside the scope of this study and requires careful control of movement type as well as the material content and location of ferromagnetic elements. We consider this an important direction for future work, which will allow a more realistic estimation of DBS-related artifacts in MEG. Furthermore, the dipolar activity used in this study was a simple 20 Hz sinusoid, which occupies a narrow frequency band. A more realistic approach would involve stimulating with a complex waveform resembling local field potentials, reconstructing it at the source level, and quantifying distortion across a broader spectrum. This approach is not currently possible with the manufacturer’s phantom, but we plan to include such an approach in future work.

We position the external stimulator outside the MSR. While this setup enables realistic characterization of DBS-induced noise in externalized lead experiments (i.e., with patients who have undergone DBS lead implantation but not yet stimulator implantation, allowing concurrent deep brain recordings), it does not account for noise originating from the generator and its ferromagnetic component materials (i.e., the second DBS artifact component), which are likely to generate low-frequency artifacts. Another potential limitation is the use of a “dry” phantom (i.e., it is not filled with saline or other conductive medium but instead uses triangular line currents to generate the dipoles [30]). To enable stimulation, we inserted the lead into a syringe filled with conductive gel. This setup constrains the current field, which may alter artifact characteristics. That said, because our experiments used bipolar stimulation—where current loops are relatively focal—the influence of medium geometry is likely less pronounced than it would be under monopolar stimulation [31].

Finally, our analysis was restricted to dipole fitting as the source reconstruction method. While convenient and well-suited for quantifying error in a single dipole, this approach does not generalize to situations with multiple simultaneous sources with complex, broad-spectrum neurophysiological signals. In the brain, many sources are active in parallel, creating highly complex spatiotemporal patterns. For this reason, distributive source imaging methods such as minimum norm estimates [32] or linearly constrained minimum variance beamforming [33] are more commonly applied in research. Future studies should therefore assess how DBS artifacts affect these distributed reconstruction approaches, possibly by incorporating a “wet” (i.e., saline-filled) phantom with the introduction of multiple complex waveforms simultaneously.

## 6. Conclusions

DBS artifacts in MEG arising directly from the stimulation current can be quantitatively characterized and do not substantially increase dipole fitting accuracy. However, further studies are needed to investigate additional potential sources of artifacts, such as the movement of ferromagnetic materials, and to evaluate the impact of DBS artifacts on distributed source reconstruction methods in MEG.

## Figures and Tables

**Table 1:** Experimental conditions.

DBS	Amplitude [mA]	Electrodes	cHPI
Off	-	-	-
On	1	Ring (0–3)	Off
On	5	Ring (0–3)	Off
On	15	Ring (0–3)	Off
On	5	Segmental (1a–1b)	Off
On	5	Segmental (1a–2a)	Off
On	5	Ring (0–3)	On

**Table 2:** Mean and standard deviation of errors across different DBS amplitudes.

		Mean Error	STD Error	*p* Value *
Location [mm]	DBS off	1.95	0.98	0.14
	DBS on 1 mA ring	1.78	1.26	
	DBS on 5 mA ring	1.69	1.63	
	DBS on 15 mA ring	1.90	1.01	
Angle [deg]	DBS off	1.92	1.10	0.56
	DBS on 1 mA ring	2.28	2.71	
	DBS on 5 mA ring	2.28	2.58	
	DBS on 15 mA ring	2.43	2.17	
Amplitude [nAm]	DBS off	5.43	2.88	0.70
	DBS on 1 mA ring	5.57	2.51	
	DBS on 5 mA ring	5.81	3.17	
	DBS on 15 mA ring	5.82	3.52	

* *p* value: Friedman test.

**Table 3:** Mean and standard deviation of errors across different DBS electrode configurations.

		Mean Error	STD Error	*p* Value *
Location [mm]	DBS on 5 mA ring	1.69	1.63	0.2725
	DBS on 5 mA 1a2a	1.55	1.32	
	DBS on 5 mA 1a1b	1.50	0.92	
Angle [deg]	DBS on 5 mA ring	2.28	2.58	0.7408
	DBS on 5 mA 1a2a	2.52	2.64	
	DBS on 5 mA 1a1b	1.95	1.96	
Amplitude [nAm]	DBS on 5 mA ring	5.81	3.17	0.6703
	DBS on 5 mA 1a2a	5.11	2.27	
	DBS on 5 mA 1a1b	5.76	1.75	

* *p* value: Friedman test.

**Table 4:** Mean and standard deviation of errors across different cHPI conditions.

		Mean Error	STD Error	*p* Value *
Location [mm]	DBS off	1.95	0.98	0.20
	DBS on cHPI off	1.69	1.63	
	DBS on cHPI on	1.57	0.88	
Angle [deg]	DBS off	1.92	1.10	0.50
	DBS on cHPI off	2.28	2.58	
	DBS on cHPI on	2.06	2.10	
Amplitude [nAm]	DBS off	5.43	2.88	0.90
	DBS on cHPI off	5.81	3.17	
	DBS on cHPI on	4.45	3.01	

* *p* value: Friedman test.

## Data Availability

The dataset is available on request from the authors.

## Supplementary Materials

The following supporting information can be downloaded at https://www.mdpi.com/article/10.3390/brainsci16060554/s1. Table S1: Phantom Positional Parameters Comparison between DBS-on and DBS-off.
