# Supplementary material for: Phantom Quantification of Magnetoencephalography Source Imaging Distortion Caused by Deep Brain Stimulation
Source: Brain Sci. 2026 May 22;16(6):554. doi: 10.3390/brainsci16060554 (PMC13297442; doi:10.3390/brainsci16060554)
Supplement: Supplementary file 1 [file brainsci-16-00554-s001.zip › brainsci-4311759-supplementary.pdf]

	DBS off		DBS on		*P-value
	mean	std	Mean	std	
Q1	0.006204899814762524	0.00032212917382376353	0.00620143095265911	0.00019753614547460122	0.88
Q2	0.002406236703811196	4.2011937528830904e-05	0.0024185582058752196	2.9100379867697813e-05	0.55
Q3	-0.07808540994319739	5.3814551457311344e-05	-0.07809266848871833	3.8853071743854986e-05	1.0
X	-0.0006406735848610304	6.5368320705051555e-06	-0.0006429153402996293	6.120983203943701e-06	0.45
Y	-0.0035440966212296043	1.213670672009022e-05	-0.0035389056752439295	8.549693963974048e-06	0.29
Z	0.004147515498936771	5.205354953262246e-06	0.00414547555651758З	3.645816355668911e-06	0.33
GOF	0.9999074994630837	1.5668507969911058e-06	0.9999084679229077	1.935270979181069e-06	0.23

*P value: Wilcoxon rank-sum statistic for two samples

Description: Mean and standard deviation of positional parameters (linear displacement and rotation) and gof for DBS-off (left) and DBS-on (right) conditions.
